# Integrative network-based Bayesian analysis of diverse genomics data

**DOI:** 10.1186/1471-2105-14-S13-S8

**Published:** 2013-10-01

**Authors:** Wenting Wang, Veerabhadran Baladandayuthapani, Chris C Holmes, Kim-Anh Do

**Affiliations:** 1 Department of Biostatistics, University of Texas MD Anderson Cancer Center, Houston, USA; 2 Department of Statistics, University of Oxford, Oxford, UK; 3 MRC Harwell, Oxon, UK

## Abstract

**Background:**

In order to better understand cancer as a complex disease with multiple genetic and epigenetic factors, it is vital to model the fundamental biological relationships among these alterations as well as their relationships with important clinical outcomes.

**Methods:**

We develop an integrative network-based Bayesian analysis (iNET) approach that allows us to jointly analyze multi-platform high-dimensional genomic data in a computationally efficient manner. The iNET approach is formulated as an objective Bayesian model selection problem for Gaussian graphical models to model joint dependencies among platform-specific features using known biological mechanisms. Using both simulated datasets and a glioblastoma (GBM) study from The Cancer Genome Atlas (TCGA), we illustrate the iNET approach via integrating three data types, microRNA, gene expression (mRNA), and patient survival time.

**Results:**

We show that the iNET approach has greater power in identifying cancer-related microRNAs than non-integrative approaches based on realistic simulated datasets. In the TCGA GBM study, we found many mRNA-microRNA pairs and microRNAs that are associated with patient survival time, with some of these associations identified in previous studies.

**Conclusions:**

The iNET discovers relationships consistent with the underlying biological mechanisms among these variables, as well as identifying important biomarkers that are potentially relevant to patient survival. In addition, we identified some microRNAs that can potentially affect patient survival which are missed by non-integrative approaches.

## Background

As the technologies for screening different types of alterations on the whole genome have been applied to oncology studies, it has been shown that cancer is a complex disease that contains many different types of genetic and epigenetic alterations, and focusing on any single type alteration will only provide an incomplete view of the cancer genome [1,2]. In order to obtain a global perspective of the cancer genome, it is essential to interrogate all types of genomic, epigenomic, transcriptomic and proteomic alterations in the cancer genome. Motivated by this rationale, The Cancer Genome Atlas (TCGA), which is a worldwide research program launched in 2006, collects data measured multiple types of cancer genomic alterations on the same set of samples for more than 20 types of cancer (http://cancergenome.nih.gov, [3]). For example, in TCGA glioblastoma multiforme (GBM) study we consider here, there are over 500 tumor samples with DNA copy number, mutation, methylation, and gene expression information available for downstream analysis.

One major goal of integration studies that combine multiple molecular platforms is to improve the understanding of complex networks of biological processes underlying cancer and subsequently, to discover how the networks affect patient clinical outcome (e.g., pathological complete response, progression free survival time). For example, gene networks can be reconstructed by integrating gene expression and genetic data [4], protein signaling pathways can be reconstructed using reverse-phase protein arrays [5], and microRNA regulated networks can be inferred by integrating microRNA and the gene expression information from their target genes's expression [6].

**Figure 1:**
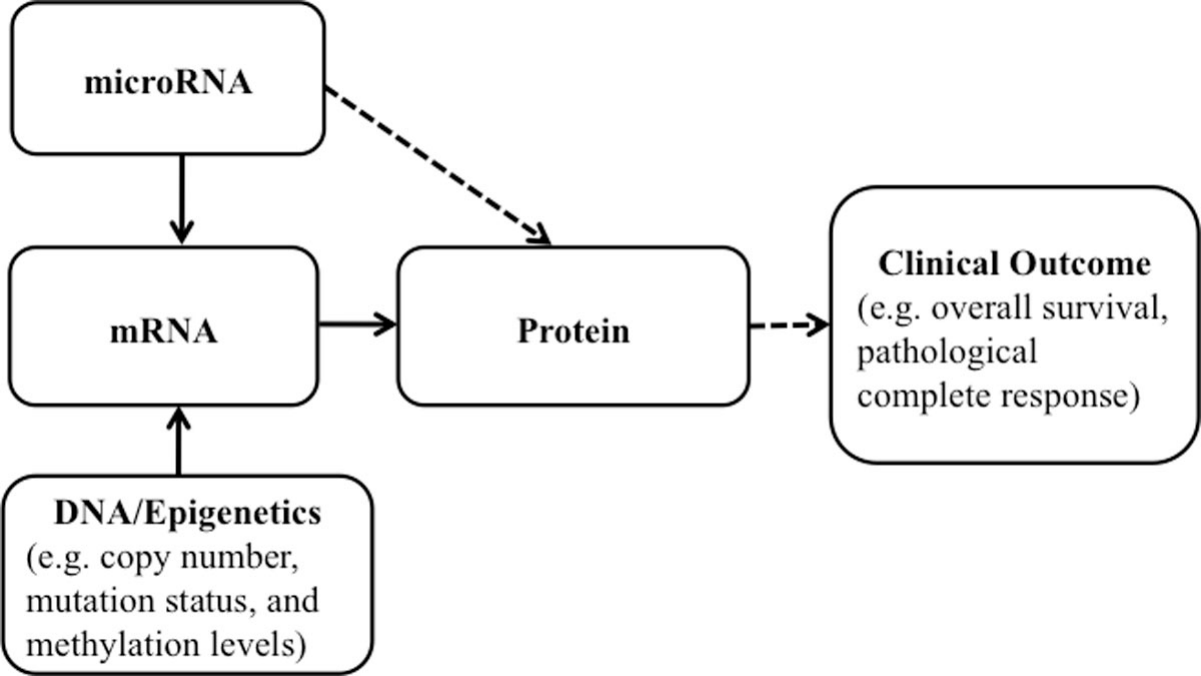
**Associations among different molecular platforms and with clinical outcome**. Solid (dashed) arrow: features from one platform are influenced directly (indirectly) by the products from the other platform.

As shown in Figure 1, the underlying biological process among different molecular features can be described as follows. The molecular features measured at the transcriptional level (e.g., messenger RNA expression) affect clinical outcomes more directly than the molecular features measured at the DNA level (e.g., copy number, methylation, and mutation status); and the molecular features related to post-translational modification (e.g. protein expression) affect clinical outcome more directly than the mRNA expression. For example, molecular features measured at the DNA level affect clinical outcome by regulating mRNA expression [7-9]. Similarly, microRNAs are post-transcriptional regulators that bind to complementary sequences on target mRNAs, usually resulting in translational repression or target degradation, which then influence clinical outcome [10].

In this article, we exemplify our methodology and focus on studying the underlying biological process among microRNA, mRNA expression, and patient-specific clinical outcomes in glioblastoma. Analyzing the relationships among other multiple molecular platforms can be done analogously (see Conclusion and Discussion). There are approximately 2000 annotated human microRNAs to date and this number is still increasing. The underlying biological relationships among microRNA and mRNA are very complicated. It has been shown that a microRNA can regulate the mRNA expression of any gene regardless of its locus, and each microRNA can have multiple target genes. The regulatory relationship between a microRNA and a gene only depends on their inherent features (e.g., microRNA sequence and structure). Currently, there are four popular algorithms - miRanda [11], TargetScan [12], PITA [13] and PicTar [14], which use the microRNA sequence and structure information to predict their target genes. However, these algorithms do not consider the effects of microRNA and gene expression on clinical outcomes.

In the following article, we first introduce an integrative network-based analysis (iNET) approach to study the relationships among features from multi-platform genomics data. In particular, we study different association networks for microRNA, mRNA expression, and patient clinical outcome. We then propose an objective Bayesian model selection approach to select these networks via Bayes factors. We generate numerical examples to show the advantage of the iNET approach compared to non-integrative approaches and to demonstrate the power gains of the iNET approach in true model selection. We apply the iNET approach to a GBM study from TCGA, and identify candidate microRNAs with potential effects on patient survival, several of which have been implicated in previous studies. In the end, we provide our conclusions and discussions.

## Integrative network-based analysis (iNET)

**Figure 2:**
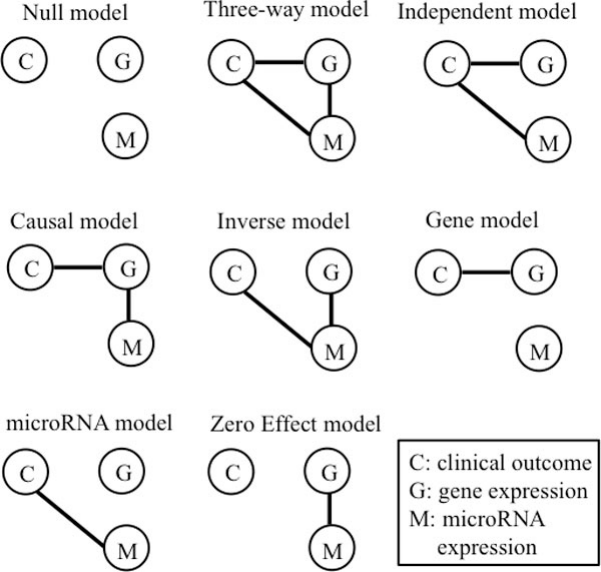
**Eight possible graphical models for each {microRNA, gene expression, clinical outcome} triplet**. An edge between any two nodes means these two nodes are dependent given the third node.

Unlike previous approaches that integrate multiple features from different genomic alterations, we take a "feature-specific" approach to model the underlying biological mechanisms. We illustrate our methodology using two platforms, with extension to multiple platforms done in an analogous manner. Let one *triplet *represent a combination of the expression levels for one gene/probe, one microRNA, and the associated patient-specific clinical outcome, which is denoted by {G,M,C}. Hence, for a given triplet there are 2^3^ = 8 possible combinations that reflect the relationships as specified below and illustrated in Figure 2:

• Null model: microRNA, gene expression and clinical outcome are independent of each other.

• Three-way model: Both gene expression and microRNA expression affect clinical outcome and moreover, the microRNA and gene expression are dependent, conditioning on clinical outcome.

• Independent model: Gene expression and microRNA expression affect clinical outcome independently. However, microRNA and gene expressions are independent, conditioning on clinical outcome.

• Causal model: microRNA expression is correlated with gene expression, which then is correlated with the clinical outcome. MicroRNA expression is not independent of clinical outcome, conditioning on gene expression. This relationship is consistent with the underlying biological mechanisms that microRNA regulates gene expression, which then affects clinical outcome.

• Inverse model: Gene expression is correlated with microRNA expression, which then is correlated with the clinical outcome. Gene expression is independent of clinical outcome, conditioning on microRNA expression. The relationship in this model is the inverse of the relationship in the causal model.

• Gene model: Only gene expression is related to clinical outcome.

• microRNA model: Only microRNA expression is correlated to clinical outcome.

• Zero-effect model: Neither gene expression nor microRNA expression is correlated with patient outcome. There is only correlation between gene expression and microRNA expression.

Out of the 8 possible graphical models, the three-way model, the independent model and the causal model reflect different underlying biological processes that are of particular interest to biomedical researchers. For example, the associations between microRNAs and clinical outcomes have been reported by other researchers [15,16], and the associations between microRNAs and their target gene expression have also been reported in [17]. The null model, inverse model, gene model, microRNA model and zero effect model also reflect meaningful biological processes but are relatively less interesting from a scientific standpoint. The goal is to survey which genes and microRNAs follow the three-way model, independent model, and causal model, respectively. To achieve this goal, we borrow the traditional approaches for studying undirected networks, Gaussian graphical models (GGM, [6,18,19]), to study the dependency structure in each triplet. However, there are substantial differences between studying traditional gene networks and our study: 1) Goals: gene network studies aim to determine the relationship of a large number of features from one molecular platform/assay in a single graph, while our study focuses on investigating the relationships among one feature from each platform for multiple platforms. 2) Scale: only one high-dimensional graph needs to be estimated for network studies, while numerous (on the order of thousands) low-dimensional graphs need to be estimated for our study. 3) Inference: the interest of network studies is to estimate the strength of the edges in the one large scale network, while the interest of our study is to determine the dependency structure among multiple molecular platforms. For example, both Stingo *et al*. [6] and our study are about integrating microRNA and mRNA expressions. In Stingo et al. study [6], 23 mouse microRNAs and 1297 potential target genes were analyzed in one graphical model. They were interested in the estimations of a large regulatory network for all these features under two different treatment conditions. In contrast, our study is focused on investigating the dependency structure of each "microRNA-gene-clinical outcome" triplet. We investigate all the possible combinations to obtain the microRNAs and genes with the relationships consistent with the real biological process.

In essence, to explore the biological relationships among different platforms, we cast the iNET approach as a model selection/comparison problem in GGMs. We use a Bayesian approach to calculate metrics that are most supported by the observed data to evaluate these different models. Specifically, we use an objective model selection procedure based on Bayes factors (e.g., see [20]) for two main reasons. Firstly, based on this approach, the Bayes factor has a closed-form expression which saves us significant computational time and cost in the analysis of such high-dimensional datasets. Secondly, this approach utilizes an automatic objective Bayesian prior for model selection; thus it eliminates the biases caused by choosing too strong or too vague priors. We now explain this procedure in detail below.

## iNET algorithm

The GGM [21] is a class of models for multivariate Normal distributions. In general, let ***x ***= (*x*_1_, *. . *. , *x_p_*)' be a *p*-dimensional Normal random vector with mean *µ *and covariance matrix Σ. In our application, *p *= 3 corresponds to the {gene,microRNA,clinical outcome} triplet detailed in the previous section. For simplicity, we assume throughout that *µ *= 0 (i.e., the data is centered). Let $\text{Ω}={\text{Σ}}^{-1}$ denote the inverse covariance, also known as the concentration matrix with elements $\omega_{ij}$. Then the partial correlation between *x_i _*and *x_j _*given all the other variables is $\rho_{ij}=-\omega_{ij}/\sqrt{\omega_{ij}\omega_{jj}}$. Thus $\omega_{ij}=0$ if and only if *x_i _*and *x_j _*are conditionally independent given all other variables.

The GGM can be represented by an undirected graph *G *= (*V*, *E*), where *V *is a set of vertices representing the variables and *E *is a set of undirected edges indicating the relationships among the variables. The graph represents the model by drawing an edge between vertices *i *and *j *when $\omega_{ij}\neq 0$. Complete graphs are defined as graphs having (*i*, *j*) *∈ E *for every *i, j ∈ V*. A graph *C *is called a *clique *if it is a maximal complete subgraph. A graph *S *is called a *separator *if it is the overlap of two cliques. We denote the sets of cliques and separators of a graph by *C *and *S*, respectively (for details see [22]). For example, in Figure 2, the cliques for the independent model are {C, M} and {C, G}, and the separator is {C}.

There are various approaches to perform model selection in the GGMs. For example, Whittaker proposed the traditional stepwise forward-selection or backward deletion approach for small sized GGMs [23]. Bayesian shrinkage approaches for large scale gene networks are described in [24-26]. Here, we follow a Bayesian approach to solve this problem for two reasons: 1) the proposed Bayesian approach provides us an automatic objective prior for the model selection, 2) the resultant Bayes factors from the proposed Bayesian approach have a closed form, hence the algorithm is computational efficient.

Suppose we observe *n *samples (***x***_1_, . . . , ***x***_n_) of *p*-dimensional vectors from an unknown decomposable graph *G*, where each ***x***_i _~ N (0, Σ), with unknown covariance matrix Σ. Let *X *be the *n × p *matrix of observed data. The posterior distribution of the graph *G *given *X *can be expressed as,

(1) π(G|X)∝π(G) ∫ π(X|Σ,G)π(Σ|G)dΣ,

where *π*(*G*) is the prior probability of the graph *G*, and *π*(Σ*|G*) is the prior for Σ given *G*.

One major problem in model selections is that the integral in equation (1) is very sensitive to different choices of the prior, and depends on sample size [22,27]. Hence, in model selection, it is critical to choose an appropriate *π*(Σ*|G*) that should at least have the two properties: a) *π*(Σ*|G*) should be a conjugate prior for practical reasons (e.g., computation efficiency); b) both improper priors and vague proper priors cannot be used since they may confound graph selections.

A conjugate prior was first proposed as *π*(Σ*|G*) ~ *HIW_G_*(*σ*, *τ I*) for a decomposable graph *G *[27], where *HIW *denotes a hyper-inverse Wishart distribution [28] and the scale matrix *τ I *is proportional to the identity matrix. The scale parameter *τ *needs to be carefully chosen to be close to the real scale of the data for this group of priors. A small scale will result in lack of discrimination among graphs we compare, and a large scale will overpower the likelihood.

An alternative construction of this prior, $\pi \left(\text{Σ}|G\right)\sim HIW_{G}\left(gn,gX^{\prime }X\right)$, was suggested in [29], where *g *is between 0 and 1. This prior is referred to as HIW g-prior, similar to Zellner's *g*-prior in linear regression [30]. It is shown that this prior corresponds to the implied fractional prior for selecting a graph using fractional Bayes factors [29] .

The fractional Bayes factor was motivated by the partial Bayes factor, which uses a part of samples to train the noninformative priors as proper priors, and the remaining data is employed to perform model comparisons [31]. It is usually applied when prior information is weak. The parameter *g *in the HIW *g*-prior can be viewed as the fraction of the likelihood used for training the noninformative prior. However, if we interpret HIW *g*-prior as the fractional Bayes factors, we cannot impose a hyper prior on *g *because *g *is no longer a model parameter. Instead, it represents the fractional power of the likelihood which is used for training the noninformative prior. Intuitively, we want to save as much of the data as possible to choose between models. Hence, we simply choose *g *= 1*/n*, equivalent to letting the training sample size to equal 1. In summary, there are several advantages to using this HIW *g*-prior: a) The conjugate property of the prior produces closed-form Bayes factors; thus the selection of thousands of models becomes feasible; b) compared to using the conventional prior (whose results depend largely on the arbitrary choice of a constant), using HIW *g*-prior (which corresponds to the implied fractional prior for (Σ*|G*)), automatically provides us an objective Bayesian approach for the model selection; c) the Bayes factors based on the HIW *g*-prior is information consistent [29].

Bayes factor calculation: Let *G*_0 _denote the null graph having no edges, and let *G_A _*denote the graph to be compared with the null. The Bayes factor for comparing these two models is

(2) $$\begin{array}{c} BF\left(G_{0}:G_{A}\right)=K\frac{\prod_{j=1}^{p}|\frac{1}{2}X_{j}^{\prime }X_{j}{|}^{\frac{n}{2}}}{\prod_{j=1}^{p}|\frac{1}{2n}X_{j}^{\prime }X_{j}{|}^{\frac{1}{2}}}\\ \;\;\;\;\;\;\;\;\;\;\;\times \frac{\prod_{C\in C}|\frac{1}{2}X_{j}^{\prime }X_{j}{|}^{\frac{\left|C\right|}{2}}\prod_{S\in S}|\frac{1}{2}X_{j}^{\prime }X_{j}{|}^{\frac{\left|S\right|}{2}}}{\prod_{S\in S}|\frac{1}{2}X_{j}^{\prime }X_{j}{|}^{\frac{\left|S\right|}{2}}\prod_{C\in C}|\frac{1}{2}X_{j}^{\prime }X_{j}{|}^{\frac{\left|C\right|}{2}}}, \end{array}$$

where  and  are the cliques and separators of *G_A _*and

$$K=\frac{\prod_{C\in C}{\text{Γ}}_{\left|C\right|}(\frac{n+|C|-1}{2})\prod_{S\in S}{\text{Γ}}_{\left|S\right|}(\frac{\left|S\right|}{2})}{\prod_{S\in S}{\text{Γ}}_{\left|S\right|}(\frac{n+|S|-1}{2})\prod_{C\in C}{\text{Γ}}_{\left|C\right|}(\frac{\left|C\right|}{2})}.$$

For the eight possible graphical models shown in Figure 2, let *G*_0 _denote the null model and *G*_1 _to *G*_7 _denote the seven network models, respectively. The algorithm for the iNET approach can be described as follows:

Step 1: We obtain the Bayes factor values for comparing the seven models with the null model by applying Equation (2) to each triplet.

Step 2: We sort *BF *(*G*_1 _: *G*_0_) to *BF *(*G*_7 _: *G*_0_) in a decreasing order; denote the sorted Bayes factors as *BF*_(1) _to *BF*_(7) _and their corresponding graphs as *G*_(1) _to *G*_(7)_

Step 3: If *BF*_(1)_*/BF*_(2) _is greater than 3 (this cutoff is determined according to the scale for interpretation of Bayes factors [32]), we conclude that this triplet supports the model *G*_(1)_. Otherwise, we conclude that there is not enough evidence to suggest a specific model.

Step 4: Given the selected model, we calculate the maximum likelihood estimates for the strength of the edges (the conditional correlations). For the three-way model, causal model, inverse model, and zero-effect model, if the strength of the edge between *G *and *M *is positive for a triplet, we filter out this triplet since it contradicts with the biological fact that the microRNAs are typically negatively associated with mRNAs.

## Simulation studies

We examine the performance of the iNET algorithm in model selection in two simulation studies. First, we assess the benefits of integrative analysis using the iNET approach against non-integrative approaches. Second, we investigate the statistical power of the iNET approach to select the true underlying graphical models. In both cases, we focus on the four models that are of particular interest to biomedical researchers - the three-way, independent, casual, and microRNA model. We aim to mimic the application dataset - the TCGA GBM data (see the Application section) as much as possible to keep our simulations realistic. Hence, for all the simulated datasets, we fix the sample sizes at 280, which equals the total number of patients we have in the TCGA GBM dataset. Moreover, we set the conditional correlations among the microRNA, gene expression and clinical outcome similar to the range of the correlations observed in the TCGA GBM data.

### Comparison to non-integrative approaches

We first show the benefits of data integration using the iNET approach over non-integrative approaches. We focus on the causal model, which is the most common relationship among the microRNA, gene expression and clinical outcome triplet. In the causal model, we vary the conditional correlation between clinical outcome and gene expression in the range - {0.2, 0.3, 0.4}, which is similar to the range of the conditional correlation between the clinical outcome and gene expression observed in the application dataset. For each of the three cases, the absolute value of the conditional correlation between gene expression and microRNA expression ranges continuously from 0 to 0.8. We generate 1000 datasets for each combination of correlations of gene expression-clinical outcome and gene expression-microRNA expression.

**Figure 3:**
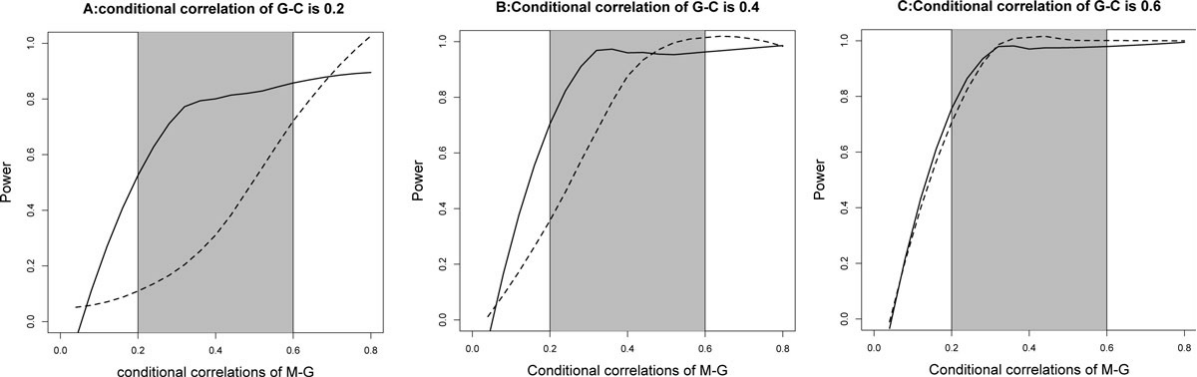
**The statistical power in identify cancer-related microRNAs by different approaches**. M: microRNA; G: gene expression; C: clinical outcome. Solid line: the iNET approach; doted line, the non-integrative approach, which evaluates the Pearson correlations between microRNA expression and patient clinical outcome; grey area: actual range of correlation in the GBM dataset in the application section.

We apply two approaches to the simulated datasets. The first approach is a non-integrative (nonINT) approach using only the microRNA expression and patient clinical outcome. In this approach, the Pearson's correlation is calculated between the microRNA expression and patient clinical outcome. The p-value from testing whether the correlation equals zero is recorded for each dataset. If the p-value is less than 0.05, we count it as a success. The second approach is the iNET approach described above and the power is calculated as the proportion (out of all datasets) when the true (i.e. causal) model gets selected. The simulation results are summarized in Figure 3. The shaded area is the the range of conditional correlation observed in the application dataset.

In Figure 3, the power of the iNET approach is substantially greater than the power of the nonINT approach when the conditional correlation of microRNA-gene expression is less than 0.4. As the conditional correlation of microRNA-gene expression increases, the power of the nonINT approach increases and become comparable with the power of the iNET approach. However, within the range of conditional consolations observed in the application dataset, the iNET approach still has greater power in identifying the correct graphical model. In summary, the iNET has greater power in identifying cancer-related microRNAs if the dependency structures for microRNAs, their target genes, and patient clinical outcome follow the causal model than approaches that ignore this information.

### Model selection

**Figure 4:**
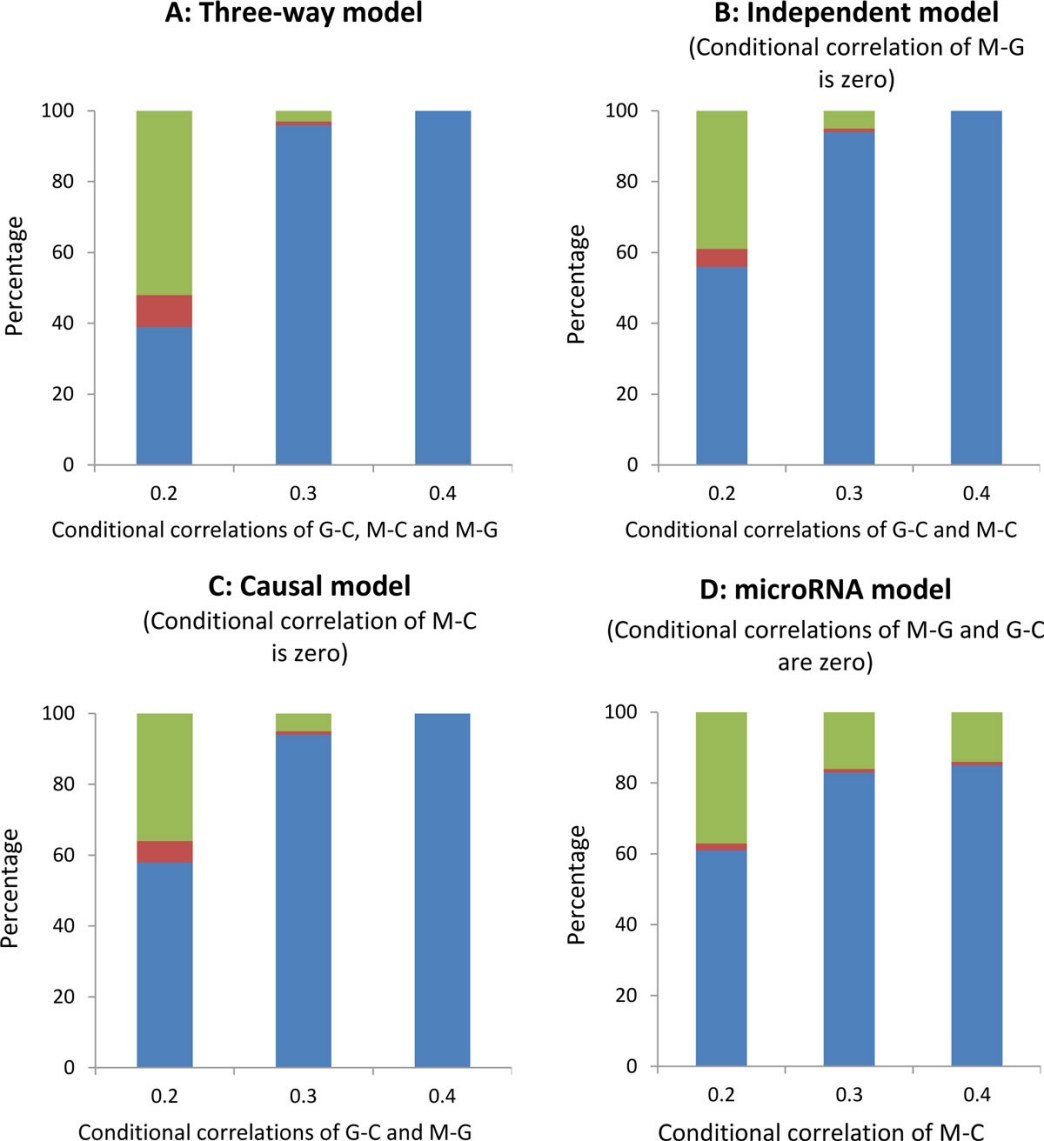
**The percentages of different models selected for different underlying models**. M: microRNA, G: gene expression, C: clinical outcome. Blue bars: percentage of the corrected model gotten selected, red bars: percentage of incorrect of models gotten selected, green bars: percentage of undecided situations since the difference between the largest Bayes factor and the second largest Bayes factor is not large enough to support the graphical model with the largest Bayes factor.

We evaluate the model selection consistency of the iNET approach in different scenarios. For each of the models considered - three-way, independent, causal, and microRNA model, we assume three different cases for the conditional correlations, i.e., the strength of the edges in the GGM. In the three-way model, we assume the conditional correlations for microRNA-gene expressions, gene expression-clinical outcome, and microRNA expression-clinical outcome are equal with values 0.2, 0.3, and 0.4. When the conditional correlations are greater than 0.4, the percentage of the true model selected by the iNET increased to 100. For each case, we generate 1000 datasets and apply our iNET algorithm. The model selection results are categorized into three groups - a) the correct graphical model is chosen, b) the incorrect model is chosen (i.e. other seven possible graphical models are chosen), and c) Undecided i.e. the BF cannot decide which model is selected since the difference between the largest BF and the second largest BF is not large enough to support the graphical model with the largest BF. We record the percentage that the correct model gets selected, the percentage that the incorrect models get selected, and the percentage of no model get selected. The result is shown in Figure 4, panel A. Similar to the the three-way model, we assume three conditional correlation cases for the independent model, causal model, and microRNA model. The results are shown in Figure 4, panels B, C, and D, respectively.

Figure 4 shows that the percentage of identifying the correct model increases as the strength of conditional correlations increase. When the conditional correlations are as low as 0.2, the iNET approach still has power that is around 0.5. Moreover, the iNET approach has a relatively low percentage of choosing an incorrect graphical model. For example, when the conditional correlations are 0.2, the iNET approach has only about 10% of times of selecting a wrong model.

Additionally, we calculate the maximum likelihood estimation for the conditional correlations for the cases that the correct model is obtained. We see that in Table 1 the mean estimation for the conditional correlation are very close to the true value. The small standard error in the model indicates that the estimations have good consistency.

**Table 1:** Evaluation of the model estimation by the iNET approach

Model	Parameters	True value	Mean estimation	Estimated error
Three-way model				

Case I	G-C/M-C/M-G	0.2	0.23	0.04
Case II	G-C/M-C/M-G	0.3	0.31	0.05
Case III	G-C/M-C/M-G	0.4	0.40	0.05

Independent model				

Case I	G-C/M-C	0.2	0.22	0.04
Case II	G-C/M-C	0.3	0.31	0.05
Case III	G-C/M-C	0.4	0.43	0.04

Causal model				

Case I	G-C/M-G	0.2	0.23	0.04
Case II	G-C/M-G	0.3	0.31	0.05
Case III	G-C/M-G	0.4	0.41	0.04

microRNA model				

Case I	M-C	0.2	0.22	0.04
Case II	M-C	0.3	0.30	0.04
Case III	M-C	0.4	0.39	0.05

M: microRNA; G: gene expression; C: clinical outcome.

In summary, from these two simulation studies, we conclude that the iNET approach can help us better understand the underlying relationship for multiple molecular features. By integrating the additional gene expression information, we gain substantial power in identifying cancer-related microRNAs. Also, we show that the iNET approach is powerful in identifying the underlying true model and in model estimation.

## TCGA glioblastoma data analysis

GBM is the most common and most aggressive malignant primary brain tumor in humans and was the first cancer type investigated by TCGA. The yearly incidence is 3 to 5 newly diagnosed cases per 100, 000 population. Most cases of GBM develop rapidly with a clinical history of only a few days or weeks. The overall median survival time for patients treated with the current standard chemoradiotherapy is approximately 15 months. The etiology of glioblastoma remains largely unknown, but epidemiology studies have shown that the risk factors for GBM include sex, age and ethnicity [33]. The TCGA GBM study includes over 500 GBM patient samples with their DNA copy number, mutation, methylation and gene expression information. All datasets analyzed here are publicly available and can be downloaded from the TCGA website. Analyzing different platforms one by one with the clinical outcome can identify the pathobiological features and molecular biomarkers in GBM. The clinical outcome we are interested in is the patient survival time after diagnosis with GBM. A ten-microRNA list was shown to play an important role in predicting GBM patient survival time [34]. The datasets used in our analysis include patient clinical features, gene expression profile and microRNA expression profile for GBM patients.

### Dataset overview

We first give an overview of the basic characteristics of these datasets and the pre-processing procedures conducted.

**Clinical characteristics**: There were 454 patients with clinical information (e.g., age at diagnosis and sex). Column 1 in Table 2 gives a brief summary of all the clinical characteristics of GBM patients. The overall survival time after diagnosis with GBM is the variable of most clinical interest. To apply the GGM procedure, we need to perform the following preliminary transformation:

**Table 2:** Patients' clinical characteristics

Characteristics	Entire patient set	Patient set for the iNET analysis
No. of patients	454	280
No. of events	349	248
Age (median)	58	57
Age (p-value from Cox model)	*<*0.0001	*<*0.0001
Age (HR from Cox model)	1.033	1.031

Patient set for GGM analysis: Reduced sample size consisting samples having data for microRNA, mRNA, and clinical outcome

• To obtain an estimated survival time for each patient, we first fit a Cox model using the only significant clinical feature in predicting patient overall survival, patient age, as the explanatory variable. Subsequently compute the Breslow estimator of the baseline hazard function.

• For each censored patient *i*, calculate the$E[\max(T_{i}^{est},T_{i}^{obs})]$, where $T_{i}^{obs}$ is the observed overall survival time and $T_{i}^{est}$ is the estimated overall survival time. These values are imputed as the actual overall survival times as if these patients were not censored.

• Log-transformation is performed for all observed survival times and imputed values.

**microRNA dataset**: The level 3 normalized microRNA data were obtained by the UNC H-miRNA 8 *× *15K array. The expression levels of 534 microRNAs were recorded for each patient in the microRNA dataset. All 534 microRNAs were considered in our model.

**mRNA dataset**: The gene expression data were obtained by using the Affymetrix Human Genome U133A chip and used the pre-processed level 2 mRNA expression data for analysis. There were 280 patient samples with all three types of information available which we use for our network-based analysis (see Table 2). We first normalized the mRNA data globally using the BrainArray CDF and RMA normalization method. After normalization, there were 12,126 measurements for each patient, and each measurement only corresponds to one gene. Since low expressing genes are subject to much greater random measurement error, genes with uniformly low expression were discarded according to the following steps.

• Divide the 280 patients into a short survival group and a long survival group using 2 years as the cutoff point (clinically meaningful in GBM). The mean expression level for each gene was calculated for the two groups, respectively.

• A gene is considered under-expressed if the mean normalized expressions for both groups are less than 5.

There are 7785 genes left after this screening step. Next, we selected the top 1000 genes most relevant to patient survival time after adjustment for patient age. This was done by fitting 7785 Cox models with age and each gene expression as predictors.

In summary, the data we used in the network analyses included 280 patients with their overall survival time, 534 microRNA expressions and 1000 gene expressions, resulting in 534 *× *1000 triplets. We are interested in identifying which of the eight graphical models in Figure 2 can best represent the relationship among the microRNA, gene, and patient survival time in each triplet.

### Analysis results

**Figure 5:**
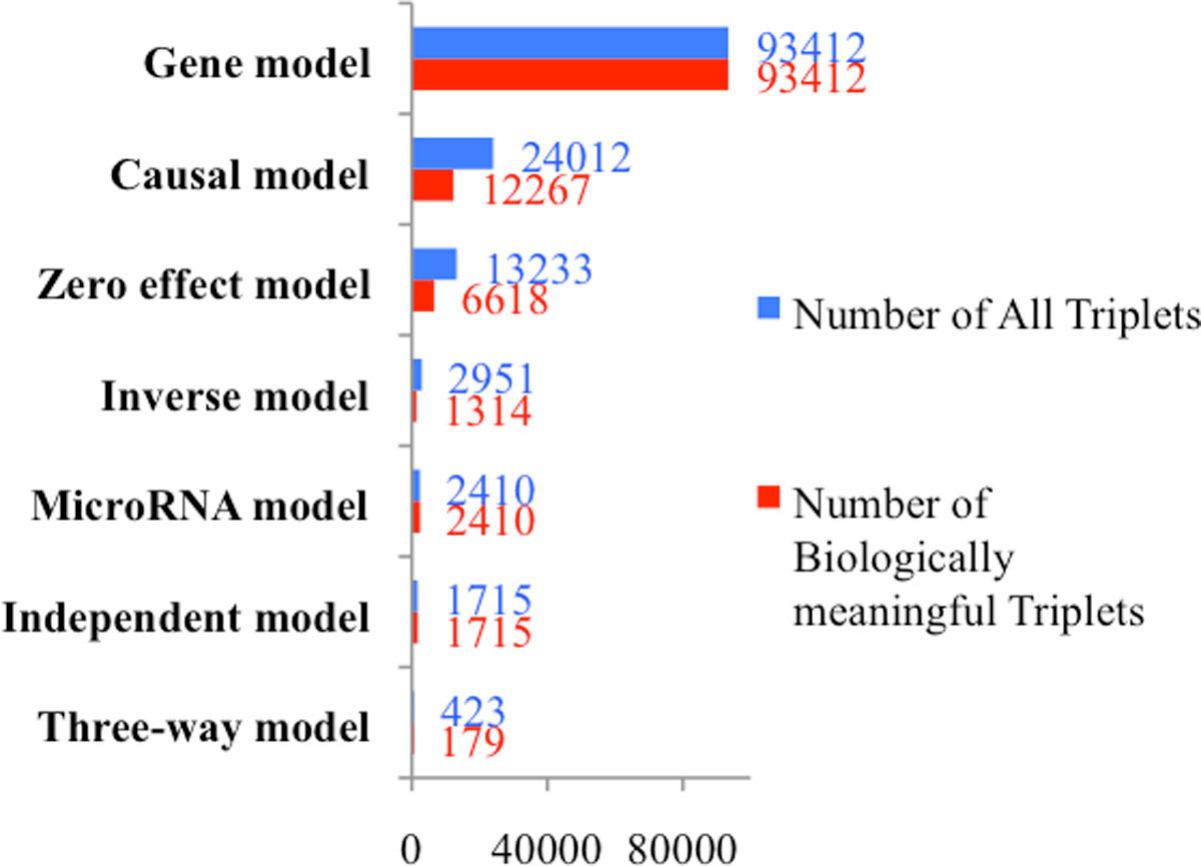
**Number of triplets in each group categorized by the network type selected via Bayes factors**.

We categorize all the triplets into eight groups according to the best fitting model indicated by their Bayes factors. The blue bars of Figure 5 summarize the number of triplets in each group. The maximum likelihood estimates are computed for the strength of the edges (the conditional correlations) for each triplet given the best graphical model that its Bayes factor indicates. If the strength of the edge between G and M is positive for a triplet, we filter out this triplet since it contradicts with the biological fact that microRNAs normally repress the expression of their target genes. Triplets that pass this filter for each graphical model are depicted by the red bars in Figure 5.

In Table 3, we summarize the number of unique microRNAs in the triplets supporting the three-way model, independent model, causal model, and microRNA model respectively. For example, there are 42 different microRNAs in the triplets supporting the three-way model. Out of the 42 microRNAs, three mircoRNAs, hsa-mir-148a, hsa-mir-221, and hsa-mir-222, are from the ten-microRNA list previously derived by [34] that can predict survival in GBM. Table 3 shows that the number is small (14) for the microRNAs involved in the group of triplets with Bayes factor supporting the independent model and the microRNA model. This indicates that although microRNA can directly affect patient survival without modulating gene expression, it is a relatively rare situation.

**Table 3:** Number of microRNAs supporting the four graphical models

Model	No. of microRNAs	Prognostic microRNAs
Three-way	42	hsa-mir-148a, hsa-mir-221, hsa-mir-222
Independent	14	has-mir-221, hsa-mir-148a, hsa-mir-222, hsa-mir-146b, hsa-mir-31
Causal	437	hsa-mir-17-5p, hsa-mir-20a, hsa-mir-106a, hsa-mir-193a, hsa-mir-146b, hsa-mir-200b
microRNA	14	hsa-mir-31, has-mir-148a, hsa-mir-221, hsa-mir-146b, hsa-mir-222

The prognostic microRNAs are obtained from [34].

**Figure 6:**
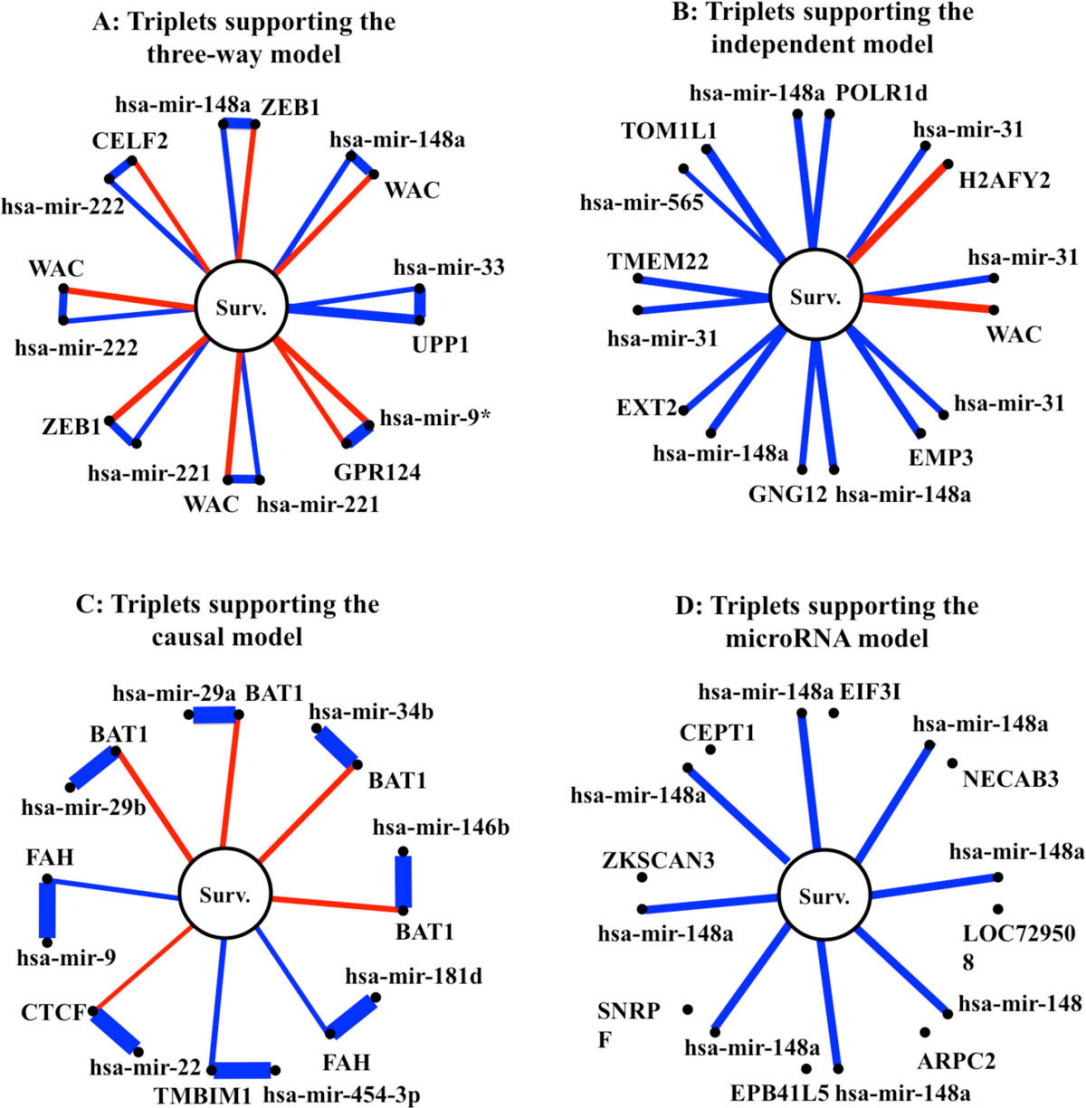
**Triplets with greatest Bayes factor supporting the three-way model (Panel A), independent model (Panel B), causal model (Panel C), and microRNA model (Panel D) compared to the Null Model Red edge: positive association; blue edge: negative association; the width of an edge: the strength of the association**.

We also plot the graphical model estimation for the 8 triplets with the greatest Bayes factor for the three-way model, the independent model, the causal model and microRNA model compared to the null model, respectively (see panels A - D of Figure 6. Many microRNAs from the ten-microRNA list are shown in these graphs. For example, Panel A of Figure 6 shows that hsa-mir-148a negatively affects patient survival through modulating different genes such as ZEB1 and WAC. In addition, it also indirectly affects patient survival negatively, which means that a higher expression of hsa-mir-148a suggests a shorter survival time. This result is in agreement with the conclusion in [34], who identified hsa-mir-148a as a risky microRNA with a hazard ratio equal to 1.21. Similarly to [34], we also identified that the hsa-mir-221 and hsa-mir-222 are risky microRNAs. These microRNAs negatively influence patient survival through modulating the expression of their target genes; they also directly affect patient survival negatively. In Panel B of Figure 6, genes and microRNAs affect patient survival independently. Analogous to findings in [34], hsa-mir-148a and hsa-mir-31 are risky microRNAs. Panel C of Figure 6 shows the microRNAs and genes consistent with the fundamental biological mechanisms. Since the microRNAs in this figure are not directly related to survival, many of them can be missed if the analysis is performed using only microRNA data [34]. In this group of microRNAs, hsa-mir-29a, hsa-mir-34b, hsa-mir-146b, hsa-mir-22, and hsa-mir-29b are risky microRNAs; while hsa-mir-181d, hsa-mir-454-3p, and hsa-mir-9 are protective microRNAs. Panel D of Figure 6 shows that hsa-mir-148a modulates patient survival negatively; while genes that are not regulated by hsa-mir-148a are not related to survival.

We employ the iNET approach as an alternative method to identify target genes for microRNAs. For the group of triplets with Bayes factors supporting the three-way model, the casual model, and the zero effect model, we can set the gene as one of the target genes of the microRNA in the triplet. We compare the targets of the 437 microRNAs determined by this approach with the targets of microRNAs in the microRNA.org database released in August 2010 (http://www.microrna.org/microrna/getDownloads.do). Target predictions on the website are based on mirSVR [35], which is a development of the miRanda algorithm. Out of the 437 microRNAs, 222 microRNAs coincide with target predictions in the database. We calculate the percentage of overlap between our target prediction and the target prediction by mirSVR method. The mean overlap percentage is 56%.

## Conclusion and discussion

In this article, we propose a network-based integrative analysis of multi-platform genomic data using microRNA, gene expression and patient survival time in a TCGA GBM data. We used an objective Bayesian model selection approach to select association networks that are most supported by the data. We found that the networks that were well represented (most supported data) are consistent with known biological mechanisms. Specifically, among the models involving microRNA, the number of triplets in the group supporting the causal model was the highest (Figure 5) and is consistent with the fundamental biological relationship that microRNA modulates gene expression, which in turn affects patient survival. Further, by integrating microRNA and gene expression information, we have a better understanding of the mechanism underlying the association between these molecular features. In addition, we identified some microRNAs that can potentially affect patient survival which are missed by analyses which do not consider this additional axis of information. Finally, we have developed freely available R-code to implement this method and is available for download under the "Software" link at the following website:

http://odin.mdacc.tmc.edu/~vbaladan/.

In this article we focused on association (undirected) networks for integrating across platforms as opposed to directed (causal) graphs. Directed graphical models, such as Bayesian networks and directed acyclic graphs (DAGs), have explicit causal modeling goals that require further modeling assumptions. Our models infer network topologies that assume a steady-state network; however, some of the gene-microRNA-clinical outcome networks may be dependent on causal relations between the nodes, which would require us to model data longitudinally to infer the complete dynamics of the network. In addition, when the number of platforms used for integrative analysis is more than 2, the number of possible biological relationships increases geometrically. For example, when the number of platforms is 4 (or 5), the number of possible biological relationships is 2^4 ^= 16 (or 2^5 ^= 32), and so on. However, only a small subset of these relationships are biologically meaningful and interpretable. In these cases, we need to use the biological knowledge as *a priori *information to reduce the relationships (hence models), and then use our framework for selection of networks from this candidate space. We leave these tasks for future consideration.

## Competing interests

The authors declare that they have no competing interests.

## Authors' contributions

VB and CCH conceived the idea. VB developed the statistical model. WW and VB designed the study and analysis. WW was responsible for software implementation, simulations and real data analysis. WW, VB and KAD wrote the manuscript. All authors read and approved the final manuscript.

## Declarations

All page charges for this article will be paid using corresponding authors' institutional funds.

This article has been published as part of BMC Bioinformatics Volume 14 Supplement 13, 2013: Selected articles from the 9th Annual Biotechnology and Bioinformatics Symposium (BIOT 2012). The full contents of the supplement are available online at http://www.biomedcentral.com/bmcbioinformatics/supplements/14/S13
